# Supplementary material for: Fatigue and Associated Factors in an Immune-Mediated Inflammatory Disease Population: A Cross-Sectional Study
Source: J Clin Med. 2022 Apr 27;11(9):2455. doi: 10.3390/jcm11092455 (PMC9099976; doi:10.3390/jcm11092455)
Supplement: Supplementary file 1 [file jcm-11-02455-s001.zip › jcm-1663035-supplementary.pdf]

Supplementary Table S1. Univariate logistic regression model for Fatigue in IBD patients

**Variables**	**OR (CI 95%)**	***p***
Age	1.02 (0.99-1.04)	0.175
BMI	1.06 (0.95-1.19)	0.295
Gender		
Male	0.42 (0.20-0.87)	**0.019**
Female	1	
Smoking		
Yes	1.02 (0.45-2.33)	0.958
No	1	
High school diploma		
Yes	0.36 (0.16-0.81)	**0.013**
No	1	
Physical Activity		
Yes	1.18 (0.59-2.34)	0.639
No	1	
Marital status, single		
Yes	1.25 (0.57-2.73)	0.582
No	1	
Occupation		
Yes	0.72 (0.37-0.1.42)	0.342
No	1	
Disease Duration	1.03 (0.99-1.08)	0.170
Disease activity		
Yes	1.01 (0.45-2.25)	0.990
No	1	
ESR	1.01 (0.99-1.04)	0.324
CRP	1.11 (0.85-1.44)	0.443
Steroids treatment		
Yes	4.92 (1.35-17.87)	**0.016**
No	1	
Biological treatment		
Yes	0.65 (0.28-1.54)	0.330
No	1	
Anxiety		
Yes	7.77 (3.49-17.28)	**4.94×10^{-7}**
No	1	
Depression		
Yes	11.33 (4.71-27.25)	**5.92×10^{-8}**
No	1	

IBD: Inflammatory Bowel Disease; OR: Odds Ratio, CI: Confidence Interval, BMI: Body Mass Index, ESR: Erythrocyte Sedimentation Rate, CRP: C-Reactive Protein

Supplementary Table S2 Univariate logistic regression model for Fatigue in IA patients

**Variables**	**OR (CI 95%)**	*p*
Age	1.01 (0.97-1.04)	0.674
BMI	1.09 (0.94-1.28)	0.256
Gender		
Male	0.22 (0.89-0.54)	**0.001**
Female	1	
Smoking		
Yes	1.08 (0.45-2.59)	0.872
No	1	
High school diploma		
Yes	0.57 (0.24-1.39)	0.220
No	1	
Physical Activity		
Yes	0.39 (0.15-0.98)	**0.045**
No	1	
Marital status, single		
Yes	0.71 (0.27-1.83)	0.477
No	1	
Occupation		
Yes	0.24 (0.10-0.59)	**0.002**
No	1	
Disease Duration	1.00 (0.94-1.07)	0.944
Disease activity		
Yes	6.64 (1.85-23.89)	**0.004**
No	1	
ESR	1.02 (0.98-1.05)	0.355
CRP	0.93 (0.79-1.10)	0.390
Steroids treatment		
Yes	1.77 (0.46-6.79)	0.405
No	1	
Biological treatment		
Yes	2.04 (0.86-4.86)	0.106
No	1	
Anxiety		
Yes	5.30 (2.12-13.26)	**3.64×10^{-4}**
No	1	
Depression		
Yes	7.59 (2.61-22.08)	**2.02×10^{-4}**
No	1	

IA: Inflammatory arthritis; OR: Odds Ratio, CI: Confidence Interval, BMI: Body Mass Index,

ESR: Erythrocyte Sedimentation Rate, CRP: C-Reactive Protein

Supplementary Table S3 Multivariable logistic regression model for Fatigue in IBD patients.

**Variables**	**OR (CI 95%)**	***p***
Age	0.99 (0.96-1.03)	0.584
BMI	1.02 (0.88-1.19)	0.764
Gender		
Male	0.88 (0.34-2.29)	0.795
Female	1	
High school diploma		
Yes	0.22 (0.06-0.77)	**0.019**
No	1	
Steroids treatment		
Yes	5.40 (1.15-25.33)	**0.033**
No	1	
Anxiety		
Yes	6.05 (2.15-16.99)	**0.001**
No	1	
Depression		
Yes	6.18 (2.30-16.60)	**2.97×10^{-4}**
No	1	

IBD: Inflammatory Bowel Disease; BMI: Body Mass Index; OR: Odds Ratio, CI: Confidence Interval

Supplementary Table S4 Multivariable logistic regression model for Fatigue in IA patients.

**Variables**	**OR (CI 95%)**	***p***
Age	0.94 (0.89-1.00)	0.038
BMI	1.56 (1.14-2.15)	**0.006**
Gender		
Male	0.22 (0.06-0.80)	**0.021**
Female	1	
Physical Activity		
Yes	0.74 (0.22-2.51)	0.623
No	1	
Occupation		
Yes	0.25 (0.08-0.85)	**0.026**
No	1	
Disease activity		
Yes	6.66 (1.37-32.40)	**0.019**
No	1	
Anxiety		
Yes	1.01 (0.25-4.17)	0.987
No	1	
Depression		
Yes	12.65 (2.28-70.25)	**0.04**
No	1	

IA: Inflammatory arthritis; OR: Odds Ratio, CI: Confidence Interval
